# Integrated Assessment of Affinity to Chemical Fractions and Environmental Pollution with Heavy Metals: A New Approach Based on Sequential Extraction Results

**DOI:** 10.3390/ijerph18168458

**Published:** 2021-08-10

**Authors:** Yuri Vodyanitskii, Dmitry Vlasov

**Affiliations:** 1 Department of General Soil Science, Faculty of Soil Science, Lomonosov Moscow State University, 119991 Moscow, Russia; yu.vodyan@mail.ru; 2 Department of Landscape Geochemistry and Soil Geography, Faculty of Geography, Lomonosov Moscow State University, 119991 Moscow, Russia

**Keywords:** heavy metals, pollution assessment, road dust, bottom sediments, soils, aerosols, particle size fractions, sequential extraction, geochemical normalization

## Abstract

To assess the affinity degree of heavy metals (HMs) to geochemical phases, many indices with several limitations are used. Thus, this study aims to develop a new complex index for assessing contamination level and affinity to chemical fractions in various solid environmental media. For this, a new integrated approach using the chemical affinity index (CAF) is proposed. Comparison of CAF with %F on the literature examples on fractionation of HMs from soils, bottom sediments, atmospheric PM_10_, and various particle size fractions of road dust proved a less significant role of the residual HMs fraction and a greater contribution of the rest of the chemical fractions in the pollution of all studied environments. This fact is due to the normalization relative to the global geochemical reference standard, calculations of contribution of an individual element to the total pollution by all studied HMs, and contribution of the particular chemical fraction to the total HMs content taken into account in CAF. The CAF index also shows a more significant role in pollution and chemical affinity of mobile and potentially mobile forms of HMs. The strong point of CAF is the stability of the obtained HM series according to the degree of chemical affinity and contamination. Future empirical studies are necessary for the more precise assessment of CAF taking into account the spatial distribution of HMs content, geographic conditions, geochemical factors, the intensity of anthropogenic impact, environmental parameters (temperature, humidity, precipitation, pH value, the content of organic matter, electrical conductivity, particle size distribution, etc.). The combined use of CAF along with other indices allows a more detailed assessment of the strength of HMs binding to chemical phases, which is crucial for understanding the HMs’ fate in the environment.

## 1. Introduction

Heavy metals (HMs), which include a wide range of chemical elements accumulating in different parts of ecosystems, may cause adverse health effects and pose a serious threat to the environment [1]. The term “heavy metals” is a loose term since it can refer to various sets of chemical elements depending on the research aim [2]. For many years, considerable attention has been focused on determining the total HMs content in soils and other solid environmental media. However, it has long been known that the total content does not accurately predict the hazard of the pollution level [3] and does not provide information on the distribution [4], mobility, and bioavailability of contaminants [5], which is associated with the HMs affinity to specific chemical fractions (which we here consider as a synonym for “geochemical phases”).

An effective method for assessing the distribution of HMs between different geochemical phases with similar chemical speciation, their level of mobility, and reactivity is chemical fractionation. This approach is based on ion-exchange reactions, the selectivity, and specificity of the chemical reagents used for fractionation, as well as the dissolution of solid-phase compounds [6]. Detailed information on HM ratios in the various geochemical phases in soils, bottom sediments, road dust, and other solid environmental media can be obtained by the multi-step sequential extraction procedure. This technique implies successive specific leaching of chemical fractions using several chemical reagents (usually 3–8 stages) applied to a solid sample. Each next reactant has greater chemical activity than the previous one, allowing the stepwise dissolution of more stable compounds [7], which are considered less mobile, bioaccessible, and environmentally hazardous of HM fractions [8]. Thus, a sequential extraction procedure provides essential information about the strength of HMs binding to particulates and the degree of their “anthropogenicity” or “geogenicity” [6], which is crucial in understanding the relationship between geochemical processes and HMs mobilization as well as the availability of HM forms for organisms and, accordingly, potential environmental risks [5,9].

To date, a large number of sequential extraction schemes have been proposed. Each of them has advantages and limitations due to the non-selectivity of the reagents and incomplete extraction of chemical forms [10,11,12,13], the number of fractionation stages, duration of laboratory manipulations, sample-reagent ratio [14,15], redistribution of HMs between phases, and risk of cross-contamination [16,17]. A detailed analysis of the limitations of various schemes for sequential extraction of HMs has been provided in some works [18,19,20].

Currently, the A. Tessier et al. [21] and the European Community Bureau of Reference (BCR) [22,23] schemes are the most commonly used sequential extraction procedures [24]. The following chemical fractions are extracted by the A. Tessier et al. method: F1—water-soluble and exchangeable, F2—carbonate, F3—reducible, F4—organic, F5—residual. To some extent, the method of A. Tessier et al. and its modifications are widely used when studying the chemical speciation of HMs in natural unpolluted soils [25], agricultural [26], urban [27], and industrial soils [28], but it is also applied for HMs fractionation in bottom sediments of rivers [29], lakes [30], seas [31], reservoirs [32], stormwater ponds [33], road dust in megacities [34] and historical centers [35], dust retained on noise barriers [36], windowsill dust [37], atmospheric aerosols [38], dry atmospheric depositions [39], fly ash of municipal solid waste [40].

The BCR method, developed for the chemical fractionation of HMs in bottom sediments, is widely used due to its reliability and reproducibility, especially for sediments in rivers [41,42], lakes [43], estuaries [44], reservoirs [45], coastal areas [46]. Unlike the A. Tessier et al. scheme, a shorter set of chemical fractions is isolated by the BCR: F1—acid-soluble (exchangeable and bound to carbonates), F2—reducible (bound to Fe/Mn oxides), F3—oxidizable (bound to organic matter and sulfides), F4—residual (metals within lithogenic minerals) fraction. BCR is also applied in some modifications to assess the ratio of HM forms in mangrove soils [47], soils in mining [9,48], industrial [49], and urban [5] areas; soils amended with waste composts or bio-sorbent materials [50,51]; airborne suspended particles [52]; river suspended sediments [53], soakaway sediments [54]; road dust in megacities [55], on various types of roads [56], and from industrial areas [57]; sewage sludge [58]; fly ash from wood biomass [59], and municipal waste incineration [10]; mining wastes [12], and ore-processing wastes [60]. For the sequential extraction of HMs from bottom sediments, the schemes according to M. Kersten and U. Förstner [61], H. Zeien and G.W. Brümmer [62], L. Leleyter and J.-L. Probst [63] are also widely used, whereas, for atmospheric aerosols, the schemes by J. Obiols et al. [64], R. Chester et al. [65], V. Zatka et al. [66], A.J.F. Espinosa et al. [67], and P. Richter et al. [68] are usually applied.

Various indices and factors are used to assess the degree of pollution intensity of mineral environmental compartments with chemical forms of HMs and the affinity of HMs to chemical phases based on sequential extraction results. Still, they have several disadvantages and limitations (an analysis is provided in the next section). Therefore, the aims of this study are as follows: (1) To develop a new complex factor for assessing contamination level and affinity to chemical fractions based on the results of HMs’ sequential extraction from solid environmental media; (2) to compare the results of calculating this new index with the traditional approach for studying chemical affinity of HMs in soils, road dust, sediments, and atmospheric particulate matter.

## 2. Assessment of Solid Components of the Environment Pollution with HMs’ Chemical Fractions

A wide range of different single-element environmental indices (calculated separately for each chemical element) and multi-element indices (integral indicators that assess environmental pollution by a set of chemical elements) are used to assess the pollution of various environments with HMs. A detailed analysis of the coefficients for assessing environmental pollution with HMs can be found in many reviews [69,70,71,72,73].

Most of the indices initially proposed to study total HMs concentrations can be used to assess environmental pollution by individual chemical fractions of HMs. However, most of them have some limitations. Thus, the pollution index (PI) [74] requires data on pre-industrial HM levels only known for a limited set of chemical elements. It also does not consider contamination with hazardous pollutants such as Sb, Mo, W, Sn, Ag, Bi, and others, which, for instance, are highly accumulated in road dust, soils, bottom sediments, suspended particles in snow, and rains in the largest megacity of Europe—Moscow [75,76,77,78]. To calculate the geo-accumulation index (Igeo) [79], the concentration factor (Cf) [80], and the enrichment factor (EF) [81], data on the background levels of pollutants are required. However, for road dust, there is no such correct background analogue. At the same time, it is also challenging for urban soils to find a comparison standard since individual horizons or the entire profile of urban soils are created or significantly geochemically transformed compared to background soils as a result of human activity. In addition, a comparison of HM concentrations with background levels can lead to a substantial overestimation of the contamination level of the urban environment with carbonate-bound HM fraction. Thus, the alkaline urban soils of Moscow located in the southern taiga are characterized by a high content of Zn and Cd in the carbonate fraction, while the presence of HMs in the carbonate fraction is not typical for the background acidic soddy-podzolic soils [82,83]. When calculating EF for chemical fractions of HMs, the use of Al, Ti, Sc, Zr, or Fe as reference elements can distort the results of pollution assessment since these elements are usually found in the most stable phases—in silicates and residual fraction [6,84,85], while HMs, actively emitted by anthropogenic sources, sharply increase their affinity to more mobile and less stable phases—exchangeable, carbonate, reducible, and organic [6,43].

To assess the HM affinity to chemical fractions a wide range of specially developed indices is used (Supplementary Materials Table S1). Among the single-element factors, the most common are: the percentage of HMs chemical fraction in the total content (%F) or percent of the sum (PFS) [86], individual contamination factor (ICF) [87], mobility factor (MF) [88], bioavailability factor (BF) [89], mobility coefficient (MC) [90], stability coefficient (SC) [90], protecting coefficient (PC) [91], anthropogenic signal index (ASI) [92], risk assessment code (RAC) [93]. Among the multi-element indices the most common are: bioavailable metal index (BMI) [94], global contamination factor (GCF) [87], global risk index (GRI) [70].

The %F or PFS index is the ratio of the HM concentration in the fraction (F_i_) to the total content of HM (C_total_):

%F = PFS = 100% × F_i_/C_total_. (1)

It is customary to denote as %F1 the percentage of the HM chemical fraction received during the first stage of the sequential extraction procedure from the total content of HM (for instance, the percentage of the exchangeable fraction in the method of A. Tessier et al. [21]), %F2—the second, %F3—the third, etc. The simplicity of the calculation and the informational content of %F determine the pervasive use of this index. The main limitations of this approach are the complexity of the comparative analysis of the results with a large number of research objects and the focus on the fractions that make the most significant contribution to the total HM content. For example, for many HMs, the %F for relatively stable, strongly bound to particles, and, therefore, low-hazard residual and organic fractions significantly exceeds the %F for the most environmentally hazardous exchangeable and carbonate fractions. Thus, even a small increase in %F for the exchangeable and carbonate fractions can pose an environmental risk. However, such an increase may be subtle (for example, an increase in the %F for the exchangeable fraction of Cd from 1% in background soils to 7% in contaminated soils). In addition, critical values for %F have not been developed (above which the metal has a chemical affinity to a specific geochemical phase and below which it practically does not have this chemical affinity).

Differences in the set of chemical fractions taken into account when calculating RAC, MF, and BF are associated with the fact that the dissolution of HM fractions can occur due to changes in the physicochemical properties of the environment, that is, RAC takes into account only bioavailable HM fractions, while MF and BF additionally consider the content of potentially mobile HM forms, the migration rate of which depends on water regime, temperature, and redox potential [9], pH value and content of carbonates [8], electrical conductivity, particle size fractions, and magnetic susceptibility [95], content of organic matter [45], intensity of the biological cycle [96]. The main limitation of ICF, RAC, MF, BF, MC, SC, and PC is non-selectivity, that is, the impossibility of defining the chemical affinity of HMs to a specific geochemical phase. ASI uses Al as a normalizing element, which has a strong affinity to silicate and residual fractions [6,85]. ASI and BMI do not allow assessing the chemical affinity of HMs to a specific geochemical phase. GCF and GRI are characterized by the same limitations as for ICF, which is used in their calculation, and also with the fact that the Tr value is proposed only for Cr, Zn, Ni, Pb, Cu, As, Cd, and Hg.

The so-called GF-analysis provide more detailed information on the HM fractionation in solid compartments of ecosystems. In this method, a bulk sample of soil, road dust, or other media is separated into its constituent particle size fractions (that is, G-analysis [97]), and then, for each G-fraction, a sequential extraction of HM chemical fractions (F-analysis) is carried out. Due to the laboriousness and complexity of interpretation of the obtained results, this approach is used not so often and is mainly devoted to the study of agricultural [98] and roadside soils [99], road dust [55,57,100], bottom sediments [101], and atmospheric aerosols [102,103]. For example, in six particle size fractions of road dust (<63 µm, 63–125 µm, 125–250 µm, 250–500 µm, 500–1000 µm, 1000–2000 µm) in the Palolo Valley, Hawaii, USA, the distribution of sequentially extracted by the BCR method acid-soluble, reducible, oxidizable, and residual fractions of Al, Zn, Pb, and Cu were analyzed [84], that is, 6G × 4F = 24 GF-fractions of each element were studied. A comprehensive analysis of the 24 fractions content for all four studied metals is challenging due to a large amount of information. Therefore, in the GF-analysis, the distribution of chemical fractions of each HM in each of the studied particle size fractions is usually investigated separately [104] or as a percentage of each GF-fraction from total HM content (conventionally, this index can be called %GF by analogy with %F) [105]. However, such approaches do not allow a comprehensive assessment of pollution by all studied HMs. The criteria for the level of environmental contamination of solid media with GF-fractions of HMs have not yet been developed.

Taking into account the limitations of the indices and factors analyzed above, one of the authors of the current article proposed a new index for assessing the affinity of chemical elements to chemical carrier phases in soils [106]:

A = (*C_phase_*/∑*C_phase_*)/*(C_soil_*/∑*C_soil_*), (2)

where *C_phase_* is the normalized HM content in the given fraction; ∑*C_phase_* is the sum of all HMs in the given fraction; *C_soil_* is the normalized content of the given HM in the initial soil; ∑*C_soil_* is the sum of all HMs in the initial soil. As for normalizing values, it was proposed to use the abundances of chemical elements in European soil, according to A. Kabata-Pendias [107]. However, the absence of a background analogue for road dust and the difficulty of choosing a correct background analogue for bottom sediments and atmospheric depositions make it necessary to apply the global HM levels as a reference standard. The average HM content in different types of soils of the world could serve as such a standard in the study of soil pollution. However, its application will impede a correct comparative assessment of contamination of the other solid environments. Therefore, to normalize the concentrations of HM chemical fractions in solid environmental media, we propose to use the average chemical composition of the upper continental crust as a reference standard, which is widely used as the global level of the content of the chemical elements [108,109]. Such normalizing will make it possible to additionally assess pollution of various environmental compartments by chemical HM forms.

In this paper, we attempted to apply a new factor for a comprehensive assessment of different environments pollution by chemical HM fractions, combining the advantages of approaches to studying the total HMs content along with the investigations of the affinity of sequentially extracted chemical fractions of HMs to various geochemical phases in solid environmental media.

## 3. New Approach for a Comprehensive Assessment of HMs Fractionation and Solid Environmental Media Pollution

### 3.1. Modification of A index

The contribution of individual chemical fractions to the total HM content is the most crucial characteristic of the pollutants speciation and, accordingly, the risk of environmental contamination, since different chemical fractions of HMs have various mobility, bioavailability, and resistance to external physicochemical and biogeochemical factors. Therefore, the new index takes into account such a contribution of fractions to the total HM content. In addition, the different contribution of HMs to the total environmental pollution requires ranking HMs following their degree of affinity to various binding sites (chemical fractions) in order to define the most actively accumulating and the most hazardous forms of HMs.

Taking into account stated above, we propose to modify the index A introduced by Yu. Vodyanitskii et al. [106]. The revised index A was called “the chemical affinity factor” (CAF) to avoid readers’ confusion. CAF is useful to assess the contamination of solid environmental media with chemical HM fractions and can be calculated by the following Equation:

CAF*_ij_* = (*NC_i_*/∑*NC_i_*)*_j-_*_fraction_/(*NC_i_*/∑*NC_i_*)_total_, (3)

where CAF*_ij_* is the chemical affinity factor of the *i*-th element to the *j*-th chemical fraction; *NC_i_* = *C_i_*/*K_i_*; *NC_i_* is the content of the *i*-th element normalized to the average composition of the upper continental crust; *C_i_* is the content of the *i*-th element in the studied object; *K_i_* is the average content of the *i*-th element in the upper continental crust; ∑*NC_i_* is the sum of *NC_i_* calculated for all studied HMs; (*NC_i_*/∑*NC_i_*)*_j-_*_fraction_ is the ratio of indicators calculated for the *j*-th chemical fraction; (*NC_i_*/∑*NC_i_*)_total_ is the ratio of indicators calculated for the total content of HM.

The numerator of Equation (3) shows the contribution of a specific chemical fraction of a particular HM to the total contamination by all HMs studied in this fraction. The denominator of Equation (3) characterizes the contribution of pollution with a particular HM to the total pollution by all studied HMs, calculated with the total HM content. Therefore, the proposed CAF index shows how close is the affinity between the pollution with the studied HMs and certain chemical fractions. In other words, how strongly the concentration of a specific chemical fraction of a particular HM increases relative to the total contamination compared with the same chemical fraction of other HMs.

Next, we consider in more detail which variables affect the CAF value. For example, we assume that the distribution of exchangeable, carbonate, reducible, organic, and residual fractions of Cd, Pb, and Zn in soils has been studied. Then Equation (3) for the exchangeable fraction of Cd takes the form:

CAF_Cd*-ex*_ = [(*C*_Cd*-ex*_/*K*_Cd_)/(*C*_Cd*-ex*_/*K*_Cd_ + *C*_Pb*-ex*_/*K*_Pb_ + *C*_Zn*-ex*_/*K*_Zn_)]/[(*C*_Cd*-tot*_/*K*_Cd_)/(*C*_Cd*-tot*_/*K*_Cd_ + *C*_Pb*-tot*_/*K*_Pb_ + *C*_Zn*-tot*_/*K*_Zn_)]. (4)

For simplicity, we denote the expression (*C*_Cd*-ex*_/*K*_Cd_ + *C*_Pb*-ex*_/*K*_Pb_ + *C*_Zn*-ex*_/*K*_Zn_) as *Z_ex_*, and the expression (*C*_Cd*-tot*_/*K*_Cd_ + *C*_Pb*-tot*_/*K*_Pb_ + *C*_Zn*-tot*_/*K*_Zn_) as *Z_tot_*. Then we get the following Equation (5):

CAF_Cd*-ex*_ = [(*C*_Cd*-ex*_/*K*_Cd_)/*Z_ex_*]/[(*C*_Cd*-tot*_/*K*_Cd_)/*Z_tot_*]. (5)

Transforming the Equation (5), we get the Equation (6):

CAF_Cd*-ex*_ = [(*C*_Cd*-ex*_/*K*_Cd_)/*Z_ex_*]/[(*C*_Cd*-tot*_/*K*_Cd_)/*Z_tot_*] = (*C*_Cd*-ex*_ × *K*_Cd_ × *Z_tot_*)/(*K*_Cd_ × *Z_ex_* × *C*_Cd*-tot*_) = (*C*_Cd*-ex*_ × *Z_tot_*)/(*Z_ex_* × *C*_Cd*-tot*_) = (*C*_Cd*-ex*_/*C*_Cd*-tot*_) × (*Z_tot_*/*Z_ex_*). (6)

According to Equation (1), *C*_Cd*-ex*_/*C*_Cd*-tot*_ is a percentage of the exchangeable Cd fraction in the total Cd content, that is, %F_Cd*-ex*_ (expressed in fractions of a unit). Taking this into account, Equation (6) takes the form:

CAF_Cd*-ex*_ = %F_Cd*-ex*_ × (*Z_tot_*/*Z_ex_*). (7)

Thus, the CAF value for a specific fraction of an individual HM is directly proportional to a percentage of this fraction in the total content of this HM. The %F index shows the affinity of HM for a particular chemical phase. In this regard, the CAF index, which is much more complex, can be considered a measure of the chemical affinity of HMs to their carrier phases.

Next, we consider in more detail the second multiplier in Equation (7), *Z_tot_*/*Z_ex_*. Obviously, this multiplier, and hence the CAF, will be maximum when *Z_tot_* is maximum, and *Z_ex_* is minimum. Let us recall the detailed notation of the multiplier:

*Z_tot_*/*Z_ex_* = [(*C*_Cd*-tot*_/*K*_Cd_ + *C*_Pb*-tot*_/*K*_Pb_ + *C*_Zn*-tot*_/*K*_Zn_)]/[(*C*_Cd*-ex*_/*K*_Cd_ + *C*_Pb*-ex*_/*K*_Pb_ + *C*_Zn*-ex*_/*K*_Zn_)]. (8)

In fact, the concentration of the exchangeable fraction of HM is the product of the total content of this HM by a percentage (expressed in fractions of a unit) of the exchangeable fraction of this HM, that is, for example, for cadmium *C*_Cd*-ex*_ = *C*_Cd*-tot*_ × %F_Cd*-ex*_. With this in mind, we get:

*Z_tot_*/*Z_ex_* = [(*C*_Cd*-tot*_/*K*_Cd_ + *C*_Pb*-tot*_/*K*_Pb_ + *C*_Zn*-tot*_/*K*_Zn_)]/[(*C*_Cd*-tot*_ × %F_Cd*-ex*_/*K*_Cd_ + *C*_Pb*-tot*_ × %F_Pb*-ex*_/*K*_Pb_ + *C*_Zn*-tot*_ × %F_Zn*-ex*_/*K*_Zn_)]. (9)

The average HM content in the upper continental crust (*K*) is a constant for each HM since it depends neither on the studied object nor on concentrations of chemical HM fractions in it. Therefore, it turns out that the expression *Z_tot_*/*Z_ex_* will be maximum when a percentage of the studied fraction (in our example, exchangeable) of each HM in the total content of each HM is minimal, that is, in our example, %F_Cd*-ex*_, %F_Pb*-ex*_, and %F_Zn*-ex*_ should be minimal. In this case, CAF for the exchangeable fraction of Cd (Equation (7)) will be maximal when %F_Cd*-ex*_ is maximal, and %F_Pb*-ex*_ and %F_Zn*-ex*_ are minimal simultaneously. CAF takes on high values when the analyzed HM has a strong chemical affinity to the studied geochemical phase. In addition, with constant %F_Cd*-ex*_, %F_Pb*-ex*_, and %F_Zn*-ex*_ values, the CAF value will increase with an increase in the total content of each of the considered HMs (Cd, Pb, and Zn). That is true since an increase in the total content of Cd, Pb, and Zn will lead to a disproportionate increase in the numerator and denominator values in Equation (9) because %F_Cd*-ex*_, %F_Pb*-ex*_, and %F_Zn*-ex*_ are always less than 1. Thus, CAF shows the affinity of HM to the chemical phase and considers the intensity of metal contamination of the studied solid environmental sample. For instance, high CAFs for the exchangeable Cd fraction in soils show that Cd has an affinity to the exchangeable phase of soils. In addition, such high CAF values denote a more significant contribution of the exchangeable Cd fraction to the exchangeable fraction of all HMs in soils than the contribution of total Cd content to the total content of all HMs in soils.

In other words, CAF > 1 indicates that the chemical phase more selectively fixes the metal compared to other HMs (and makes a significant contribution to the contamination of the studied object with this fraction of HM). On the contrary, at CAF < 1, this metal is more strongly “rejected” by the chemical phase than other HMs. For example, CAF > 1 for the carbonate fraction of Cd in soils indicates that the carbonate-bound Cd makes a more significant contribution to the total soil contamination with the carbonate fraction of all studied HMs than the contribution of the total Cd content to the pollution with the total content of all studied HMs. Thus, we believe that CAF is a convenient indicator not only of the chemical affinity of HMs to the geochemical fraction but also of contamination with these HMs of the studied object.

Variations in HM concentrations and the accuracy of most analytical methods (about ±20%) require the proposal of more detailed gradations of CAF. Therefore, we suggest considering the values 1 ± 0.2 as the moderate CAF level. The rest of the CAF levels could be defined by multiplying (with an increase in the CAF) and dividing (with a decrease in the CAF) the upper and lower values of moderate CAF gradation by two, respectively:

- CAF < 0.4 corresponds to an extremely low chemical affinity of HM to this geochemical phase and contamination of studied solid environmental media with this HM fraction;
- 0.4 < CAF ≤ 0.8 amounts to a low chemical affinity and contamination;
- 0.8 < CAF ≤ 1.2 equals to a probable chemical affinity and contamination;
- 1.2 < CAF ≤ 2.4 points out the clear chemical affinity and contamination;
- CAF > 2.4 corresponds to an extremely strong chemical affinity of HM to this geochemical phase and contamination of studied solid environmental media with this HM fraction.

Ranking CAF for different fractions of one HM makes it possible to identify the most polluting chemical fraction, that is, which fraction of a particular HM makes a greater contribution to ecosystem contamination. In turn, ranking the CAF for one fraction of different HMs allows identifying metals that most pollute the ecosystem with this chemical fraction. Finally, ranking CAF for different fractions of all studied HMs reveals the most dangerous chemical fraction of the most hazardous HM.

An obvious disadvantage of CAF is that the value of the factor depends on the set of HMs taken into account since in the numerator and denominator of Equation (3) HMs are normalized in relation to the sum of all HMs. Therefore, the inclusion of a larger number of HMs in the analysis can lead to a change in the ratio *NC_i_*/∑*NC_i_*. However, the advantage of CAF is that the ranked CAF series is stable. Although the HMs added to the calculations will change the CAF values themselves, these HMs will “enter” the already obtained ranked series, and the previously identified patterns will not be violated (for more details, see Section 3.2). The addition of new HMs to the analysis makes it possible to clarify the previously obtained series and determine whether the new HMs are more or less polluting agents than the already analyzed HMs. This CAF advantage can probably be effective when using single parallel extraction of HMs: the expansion of the analysis by adding new parallel extractants will make it possible to compare the assessment of contamination by new chemical forms of HMs with previously obtained data.

Further, examples of calculating CAF for chemical fractions of HMs extracted from soils, bottom sediments, and atmospheric particulate matter (F-analysis), as well as extracted from individual particle size fractions of road dust (GF-analysis) are analyzed, and the obtained data are compared with the results of assessing the chemical affinity of HMs by the widely used %F index. Four types of research objects were necessary to check the possibility of using the CAF calculation for different environments. The selection criteria for the objects were the following: (1) The possibility of applying sequential extraction and the availability of detailed published data on the chemical composition of such objects; (2) the possibility of geochemical relationships formation between the selected objects (for instance, between soils, road dust and atmospheric depositions within the urban environment [110]). Therefore, soils were selected as one of the most studied objects with a large number of publications on the sequential extraction of HMs. Bottom sediments were also chosen due to a large number of publications, and in addition, because sediments could include soil particles due to the watershed erosion. The third object is atmospheric particulate matter, which can be an important source of material for soils and bottom sediments after precipitation from the atmosphere. The last object is road dust, which is geochemically associated with soils due to soil particles’ resuspension and the atmosphere due to particulate matter deposition.

When calculating CAF, the following average contents of chemical elements in the upper continental crust were used [108], mg/kg: Ba, 628; Cd, 0.09; Co, 17.3; Cr, 92; Cu, 28; Fe, 39180; Mn, 774; Mo, 1.1; Ni, 47; Pb, 17; Sc, 14; Sr, 320; U, 2.7; Zn, 67. All calculations were based on data from specific literature examples on the content of chemical forms of HMs in solid environmental media. When choosing publications for the core data, we followed several requirements: (1) The publication should provide initial data on the content of each chemical fraction of HMs; (2) if possible, the paper should be recently published (after 2017); (3) as many metals as possible should be analyzed; (4) different metal fractionation schemes should be used for different environments. The following studies were selected: for soils, by Y. Li et al. [86]; for bottom sediments, by X. Gao et al. [111]; for aerosols, by R. Jan et al. [102]; for particle size fractions of road dust, by A. Jayarathne et al. [100]. For bottom sediments, the publication of 2010 was chosen since the content of 12 chemical elements was analyzed there, while usually only 3–6 elements are studied in such kinds of papers.

The calculations were carried out in MS Excel (Microsoft, USA); the Spearman’s rank correlation coefficients (*r_s_*) between the CAF and %F values for each HM with significance level check were calculated using the STATISTICA 8 (Dell, Round Rock, TX, USA). The *r_s_* values were calculated due to the small sample size (3–5 fractions). In addition, the Pearson’s correlation coefficients (*r*) between CAF and %F were calculated for the case when two series of variables were compared: CAF values for each fraction of each HM and similar %F values, which was made possible by increasing the sample size (number of fractions multiplied by the number of HMs). To compare the differences in the HM associations formed according to the results of %F and CAF, the cluster analysis based on %F and CAF values was performed using the STATISTICA 8 with Euclidian distances as a measure of similarity, and complete linkage amalgamation rule to distinguish stable associations of HMs.

### 3.2. Assessment of Soil Contamination with Chemical Fractions of HMs

CAF calculations for soils were performed based on the results of sequential extraction of Cd, Ni, Pb, Zn, Cr, Cu, and Mn by the A. Tessier method [21] from 44 samples of surface (0–20 cm) horizons of agricultural soils from Dongdagou stream basin in Baiyin City, Gansu province, China [86]. The following chemical fractions of HMs have been studied: F1—exchangeable, F2—carbonate, F3—reducible, F4—organic, F5—residual.

**Figure 1:**
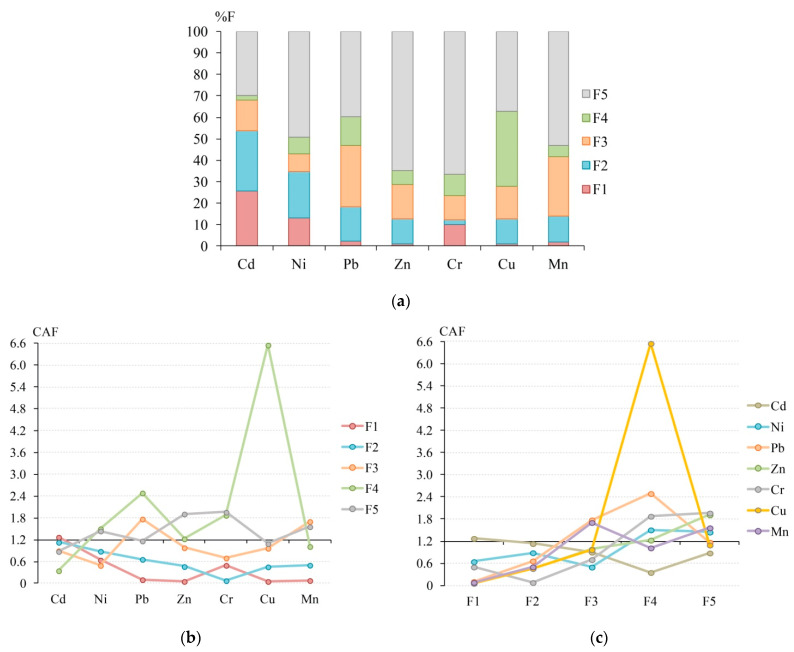
The fractionation distribution of HMs (**a**), levels of chemical affinity factor (CAF) for HMs (**b**), and levels of CAF for chemical fractions (**c**) in agricultural soils from Dongdagou stream basin, China—calculated from the data of Y. Li et al. [86]. F1 is exchangeable, F2 is carbonate, F3 is reducible (Fe-Mn oxyhydroxides), F4 is organic, F5 is residual fraction.

The initial data on the concentration of HM fractions and the value of %F for each HM fraction and the CAF values are given in Table 1 and Figure 1. As for %F, all HMs are mainly associated with the F5 fraction, followed by the F4 fraction for Cu, F3 for Pb, Zn, Cr, Mn, and F2 for Cd and Ni (Table 1, Figure 1). The F1 fraction for Cr, Ni, and especially Cd also significantly contributes to the total content of these metals in soils.

The CAF showed a more significant role of F4 and F1 and a less pronounced contribution of other fractions to the HMs content (Table 1). In contrast to %F, an extremely strong (CAF > 2.4) and clear (CAF 1.2–2.4) affinity to F4 was revealed not only for Cu, but also for Pb, Cr, Ni, and Zn; as well as for Cd to F1; for Pb and Mn only to F3; and for Cr, Zn, and Mn to F5. A series of decreasing ∑CAF (the sum of the affinity levels of all HMs of a specific chemical fraction) values well reflect a significant increase in chemical affinity of HMs to the organic phase and a decrease in the contribution of the residual phase of HMs: F4 (15.0) > F5 (10.0) > F3 (7.55) > F2 (4.21) > F1 (2.74). A rather large contribution of F3 to the content of HMs is often revealed for soils and bottom sediments [112]. The main reason for the difference between CAF and %F results is that CAF considers not only the chemical affinity but also the level of contamination with chemical fractions. In other words, %F takes into account only the contribution of the particular chemical fraction to the total HMs content, while CAF additionally shows the normalization relative to the global geochemical reference standard and contribution of an individual element to the total pollution by all studied HMs.

According to CAF, Pb, Zn, Cu, and Mn have an extremely low chemical affinity to F1, Cr to F2, and Cd to F4, which, accordingly, indicates a low intensity of soil contamination with these HM fractions. Low chemical affinity to F1 was found for Ni and Cr, to F2—for Pb, Zn, Cu, and Mn, and to F3—for Ni and Cr. The *r_s_* value between CAF and %F is significant at *p* < 0.05 only for Cu (0.9). For other HMs, *r_s_* are insignificant and equal to 0 for Ni, 0.3 for Cd and Pb, 0.7 for Zn and Cr, and 0.8 for Mn (Supplementary Materials Table S2), proving different estimates of the HMs’ affinity to specific fractions using these two various factors. The revealed differences in the assessment of %F and CAF are also confirmed by the fact that the stable metal associations identified by cluster analysis for both indices differ (Supplementary Materials Figure S1). Thus, for %F, associations are distinguished with the matching contribution of chemical fractions to the total content of the following HMs: Cd–Ni, Pb–Mn, Zn–Cr. According to the CAF, the stable associations of Cr–Ni, and Zn–Mn are formed. However, the common thing for %F and CAF is the non-association of Cu with other HMs, indicating significant differences in the fractionation of Cu compared to other HMs (primarily due to a high share of F4 in the total content and also an extremely high CAF).

For individual HMs, the main differences in assessing the chemical affinity using %F and CAF are as follows. For most HMs, CAF significantly reduces the role of F5 in comparison with the %F index, which leads to a shift of the residual fraction in a series of decreasing CAF and %F values from the first place to the second for Mn, Ni, and Cu, to the third place for Pb, and to the fourth place for Cd. For Zn and Cr, F5 remains the leading one in the total content of HMs in soils. For Cd, the CAF shows an increase in the contribution of F1 and a decrease in the contribution of F2 (fractions change places in a series of decreasing CAF values relative to a series of reducing %F values). For Mn, an increase in F4 and a decrease in F2 were found. A sharp increase in the organic and a decrease in the carbonate, exchangeable, and reducible fractions was revealed for Ni. For Pb and Zn, an increase in F4 and a decrease in F3 and F2 were found. For Cr, an increase in F4 and a decrease in F3 and F1 were investigated (Table 1).

The ranked HM series by CAF values in each chemical fraction is identical to the ranked HM series by %F values in the same chemical fraction. That is, Spearman’s correlation coefficient is 1, since according to Equation (7) CAF_Cd*-ex*_ = %F_Cd*-ex*_ × (*Z_tot_*/*Z_ex_*), CAF is directly proportional to %F, and (*Z_tot_*/*Z_ex_*) is the same for all HMs of one chemical fraction (in our example, for the exchangeable fraction). However, %F does not allow compiling a series of HMs according to the intensity of chemical affinity for all studied fractions at once, since the sum of %F is equal to 100% for each HM. In contrast, the series by the CAF value shows the change in the contribution to the total pollution. For agricultural soils from the Dongdagou stream basin, the ranking of HMs by CAF(∑F) value (the sum of CAF for all fractions of a particular HM) showed a decrease in their contribution to the total pollution in the series: Cu (9.14) > Pb (6.22) > Cr (5.13) > Ni (4.99) > Mn (4.88) > Zn (4.66) > Cd (4.55).

Using this example, we can consider the influence of the set of analyzed HMs on the results of CAF calculating. As the number of elements decreases due to the removal of Mn, the series of decreasing CAF(∑F) values change to as follows:

Cu (9.15) > Pb (6.22) > Cr (5.13) > Ni (4.99) > Zn (4.67) > Cd (4.55).

When calculating CAF for six chemical elements, the overall HMs sequence did not change, which indicates the stability of pollution assessment results. With a further reduction in the set of elements, the series will be stable even if elements with a high CAF are removed from the calculations:

Pb (7.51) > Cr (6.10) > Ni (5.73) > Zn (5.30) > Cd (4.63);

Pb (7.53) > Ni (5.74) > Zn (5.32) > Cd (4.63);

Pb (7.85) > Ni (5.94) > Cd (4.62).

As noted above, the advantage of CAF over other indicators for assessing the chemical affinity of HMs is also the fact that CAF allows identifying the most hazardous HM fraction that contaminates the studied environmental compartment the most. The full ranking of chemical fractions by CAF value showed that the highest pollution of agricultural soils from the Dongdagou basin is formed by F4 Cu (6.55) > F4 Pb (2.49) > F5 Cr (1.96) > F5 Zn (1.91) > F4 Cr (1.88) > F3 Pb (1.78) > F3 Mn (1.71) > F5 Mn (1.56) > F4 Ni (1.51) > F5 Ni (1.45) > F1 Cd (1.28) > F4 Zn (1.23). Other HM fractions have a less significant affinity for contamination with CAF < 1.2. The *r_s_* value between the series of CAF values for each fraction of each HM and the corresponding %F values is significant at *p* < 0.05 and equals 0.696, which generally indicates the presence of similar trends in the change in CAF and %F in the soils of the Dongdagou stream basin. At the same time, the Pearson’s correlation coefficient *r* between these indicators is also significant at *p* < 0.05 and equals 0.464; that is, the determination coefficient *r*^2^ is 0.215, which, taking into account Equation (7), may indicate that 21.5% of the CAF variability is associated with a change in %F (chemical affinity), while 78.5% may be due to affinity to contamination.

Thus, CAF, in contrast to %F, showed a less significant affinity to the residual fraction of all HMs, and at the same time, a more significant affinity of Cu, Pb, Cr, Ni, and Zn to the organic fraction, the affinity of Cd to the exchangeable fraction, and the affinity of Pb and Mn to the reducible fraction. The calculation of *r_s_* confirmed a great difference in the estimates of the HMs’ affinity to specific chemical fractions using CAF and %F.

### 3.3. Assessment of Bottom Sediments Contamination with Chemical Fractions of HMs

CAF calculations for bottom sediments are based on the results of sequential extraction of Ba, Cd, Co, Cu, Mn, Mo, Ni, Pb, Sc, Sr, U, and Zn by the BCR method modified by G. Rauret et al. [23] from nine samples of undisturbed surface (0–5 cm) horizons of the Daya Bay sediments in the northern South China Sea located in the eastern coast of Guangdong Province, southern China [111]. The following chemical fractions of HMs have been studied: F1—acid-soluble, F2—reducible, F3—oxidizable, F4—residual.

**Figure 2:**
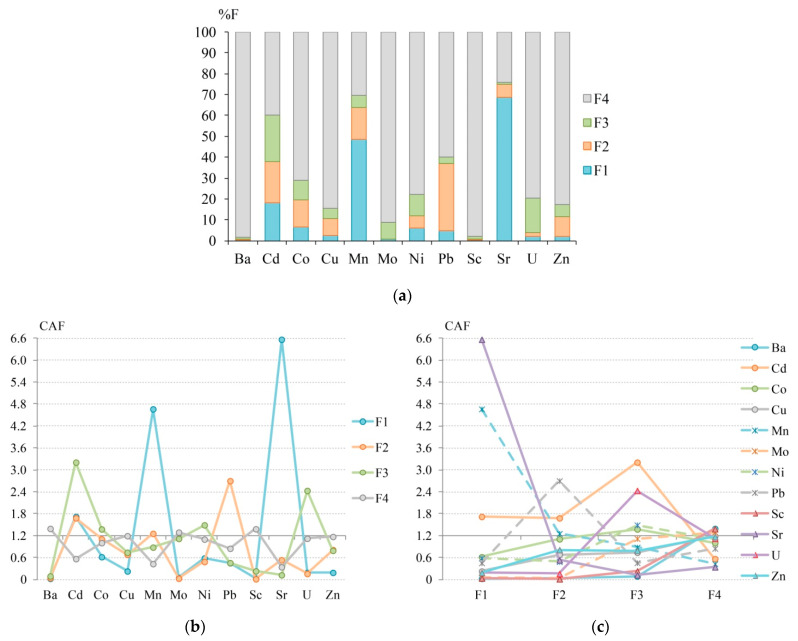
The fractionation distribution of HMs (**a**), levels of chemical affinity factor (CAF) for HMs (**b**), and levels of CAF for chemical fractions (**c**) in surface sediments of Daya Bay—calculated from the data of X. Gao et al. [111]. F1—acid-soluble (exchangeable and bound to carbonates), F2—reducible (bound to Fe/Mn oxides), F3—oxidizable (bound to organic matter and sulfides), F4—residual (metals within lithogenic minerals) fraction.

The initial data on the concentrations of HM fractions and the %F values for each HM fraction and the CAF values are shown in Table 2 and Figure 2. As for %F, the F1 fraction is the main contributor to the total Sr and Mn content in the bottom sediments of Daya Bay; for other HMs, the leading phase is F4, which is usually represented by aluminosilicate minerals [113]. Bottom sediments are characterized by a significant contribution of F1 to the total Mn content. Fraction F1 makes a noticeable contribution (>10%) to the content of Cd, fraction F2—to the content of Pb, Cd, Mn, Co, and Zn, fraction F3—to the content of Cd, U, and Ni. A significant role in the fixation of Cd was found for F1, which is probably associated with weak electrostatic adsorption of metal and an affinity for carbonates [114].

In contrast to %F, the CAF coefficient shows a less significant role of F4 in the HM accumulation: Ba, Mo, and Sc have a clear chemical affinity (CAF 1.2–2.4) for this phase. At the same time, CAF, similarly to %F, indicates an extremely strong (CAF > 2.4) affinity of Sr and Mn to F1, Pb to F2, Cd and U to F3, as well as a clear affinity of Cd to F1, Cd and Mn to F2, Ni and Co to F3. In turn, Ba, Cu, Mo, Sc, U, and Zn have an extremely low affinity (CAF < 0.4) to F1 phase, Ba, Sc, and Sr to F3, and Sr to F4, which indicates low pollution of bottom sediments with these chemical fractions. A significant increase in the role of acid-soluble and oxidizable phases with a simultaneous decrease in the contribution of the residual fraction reflects the series of decreasing ∑CAF values: F1 (15.3) > F3 (12.9) > F4 (11.8) > F2 (9.50).

The *r_s_* values between CAF and %F are 1.0 for Ba, Mo, Sc, and Zn, which indicates close estimates of the affinity of HMs for specific chemical fractions using two different coefficients (Supplementary Materials Table S2). For other HMs *r_s_* decreases from 0.8 (Cu, Ni, Sr, U) to 0.6 (Pb), 0.4 (Mn), 0.2 (Co), and −0.4 (Cd). The cluster analysis results show that stable associations of Ba–Sc, Mn–Sr, Cu–Zn, and Cd–Pb are formed by both %F and CAF, which indicates quite similar results for two indices (Supplementary Materials Figure S1). However, according to %F, Ni forms an association with U, while according to CAF, this metal forms an association with Co, which is a result of an increased share of F3 and a reduced share of F2 in the total content of U as compared to Co and Ni, but at the same time with a high CAF for F3 for U relative to Co and Ni.

For Zn, Sc, Mo, and Ba, the series of decreasing %F and CAF(∑F) values are identical, that is, using CAF to assess the chemical affinity for these HMs gives similar results as the %F-approach. Consequently, the *r_s_* value between CAF and %F for Zn, Sc, Mo, and Ba is 1.0. For other chemical elements, the affinity series for CAF and %F differ, manifested in a sharp decrease in the contribution of F4, an increase in the contribution of other fractions for Cd. In addition, a shift of F4 in this series from the first place for %F to the fourth for CAF, as well as for Co from the first place to the third, for Mn from the second place to the fourth, for Ni, U, and Pb from the first place to the second is revealed. For Cd, CAF shows an increase in the contribution of the F1 and a decrease in the F2 (fractions swapped in the decreasing series for the contribution of fractions to the total content for CAF relative to %F). For Co and Cu—an increase in F3 and a decrease in F2, for Pb—an increase in F3 and a decrease in F1 were found (Table 2). The greatest difference in the assessment of the HMs’ affinity to chemical fractions using CAF and %F was found when calculating CAF for Ni and U for which the F3 becomes the leading fraction, as well as for Pb the F2 becomes the main fraction. For this reason, the *r_s_* value between CAF and %F decreases in the following order: Cu, Ni, Sr, U (0.8) > Pb (0.6) > Mn (0.4) > Co (0.2) > Cd (–0.4). For bottom sediments of Daya Bay, ranking of elements by the CAF(∑F) value showed a decrease in contribution to the total pollution in the series: Sr (7.56) > Mn (7.23) > Cd (7.18) > Pb (4.45) > Co (4.10) > U (3.90) > Ni (3.66) > Zn (2.96) > Cu (2.84) > Mo (2.49) > Sc (1.66) > Ba (1.55).

A full ranking of chemical fractions by CAF value showed that the highest pollution of the bottom sediments of Daya Bay is formed by F1 Sr (6.57) > F1 Mn (4.66) > F3 Cd (3.21) > F2 Pb (2.70) > F3 U (2.43) > F1 Cd (1.72) > F2 Cd (1.68) > F3 Ni (1.49) > F4 Ba (1.39) > F4 Sc and F3 Co (1.38) > F4 Mo (1.29) > F2 Mn (1.26); other HM fractions have less significant affinity for contamination with CAF < 1.2. The *r_s_* value between the series of CAF values for each fraction of each HM and the corresponding %F values is significant at *p* < 0.05 and amounts to 0.804, which generally indicates similar estimates of the chemical affinity of HMs for different geochemical phases in the bottom sediments of Daya Bay when using CAF and %F. At the same time, the Pearson’s correlation coefficient *r* between these values is also significant at *p* < 0.05 and amounts to 0.417, that is, the coefficient of determination *r*^2^ is 0.174, which may indicate that a 17.4% of CAF variability is associated with a change in chemical affinity, while 82.6% can be associated with a change in the level of pollution.

Thus, the CAF index compared with %F shows a significant increase in acid-soluble and oxidizable fractions and a less significant role of the residual fraction in the accumulation of HMs, which indicates more hazardous contamination of bottom sediments with HMs when using CAF compared to %F. Calculations of *r_s_* between CAF and %F showed similar estimates of the chemical affinity of Ba, Mo, Sc, and Zn using both indices, but a great difference in the estimates for Cu, Ni, Sr, U, Pb, Mn, Co, and Cd. This difference is most clearly manifested in a sharp increase in the contribution of F3 for Ni and U and F2 for Pb when using CAF compared to %F.

### 3.4. Assessment of Atmospheric Particulate Matter Contamination with Chemical Fractions of HMs

**Figure 3:**
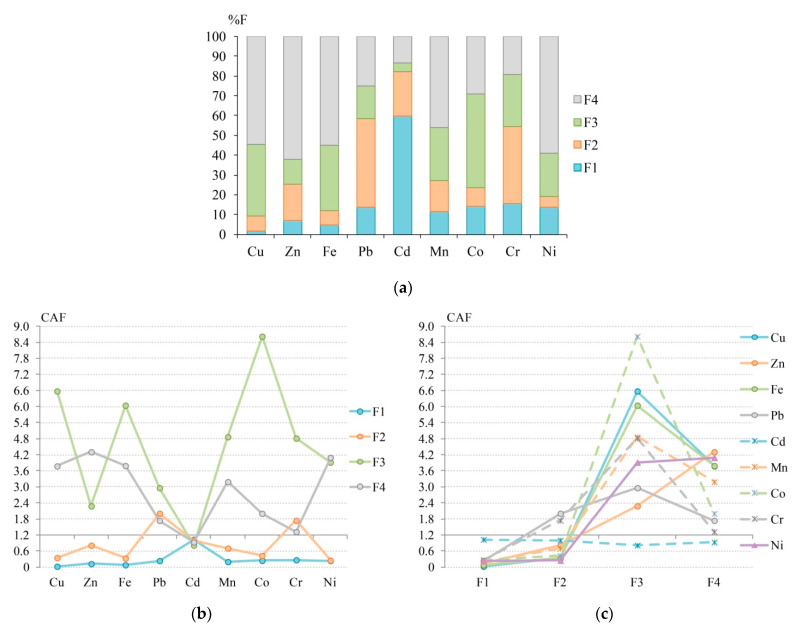
The fractionation distribution of HMs (**a**), levels of chemical affinity factor (CAF) for HMs (**b**), and levels of CAF for chemical fractions (**c**) in atmospheric PM_10_ of Pune city—calculated from the data of R. Jan et al. [102]. F1—water-soluble, F2—environmentally mobile (exchangeable), F3—the sum of carbonate and bound to oxides fractions, F4—the sum of organic and residual fractions.

CAF calculations for aerosols are based on the results of sequential extraction of Cu, Zn, Fe, Pb, Cd, Mn, Co, Cr, and Ni by the method of R. Chester et al. [65] modified by P. Richter et al. [68] from 108 samples of the coarse fraction of aerosols with a diameter 10 μm and less (PM_10_), at the roof of a building (12 m above the ground) at Hadapsar, in the eastern part of Pune metropolitan city, State of Maharashtra, India [102]. The following chemical fractions of HMs have been studied: F1—water-soluble, F2—environmentally mobile (exchangeable), F3—the sum of carbonate and bound to oxides fractions, F4—the sum of organic and residual fractions. The initial data on the concentration of HM fractions and the %F values for each HM fraction, as well as the CAF values, are shown in Table 3 and Figure 3.

As for %F, the leading carriers of Cu, Zn, Fe, Mn, and Ni in atmospheric PM_10_ in Pune are the organic and residual fraction F4, the main carrier of Cd is water-soluble fraction F1, and carrier of Co is carbonate and bound to oxides fraction F3, as well as of Pb and Cr is exchangeable fraction F2 (Table 3, Figure 3).

The CAF index also defines a significant role of F4 in the accumulation of HMs and indicates a significant role of F3, which proves close estimates of the affinity of HM to specific chemical fractions using CAF and %F. High values of *r_s_* between them confirm the similar patterns in such estimates for the two indices for Cd (1.0), Cu, Zn, Fe, Mn, Co, Cr, and Ni (0.8). The highest differences between estimates of affinity to fractions using CAF and %F has Pb, for which *r_s_* is 0.4 (Supplementary Materials Table S2). The cluster analysis results showed the formation of stable associations of Cu–Fe and Pb–Cr both using %F and CAF (Supplementary Materials Figure S1). The similarity of the results for both indices is also evident in the non-association of Co with other HMs due to a high share of F3 in the total metal content and a very high CAF for F3. However, clustering of HMs by %F shows a still stable association of Zn-Ni, while by CAF, Zn forms an association with Cd and Ni associates with Mn due to a relatively high CAF value for F3 and F4 of these HMs.

According to CAF, Cu, Zn, Fe, Mn, and Ni have an extremely strong affinity to F4 (CAF > 2.4), as well as all HMs, except for Cd and Zn, have an extremely strong affinity to F3. Pb and Cr have a clear chemical affinity (CAF 1.2–2.4) to F2, Zn—to F3, and Pb, Cr, and Co—to F4. In turn, all HMs, except for Cd, have an extremely low affinity (CAF < 0.4) to F1, and Cu, Fe, and Ni—to F2, that is, the pollution with atmospheric PM_10_ by these fractions is low. It can be seen that no HMs have CAF < 0.4 in carbonate and bound to oxides fractions, as well as in organic and residual fractions, which indicates the formation of a high pollution level of atmospheric PM_10_ with these compounds in the city of Pune. High levels of ∑CAF confirm the significant role of F3 and F4 in pollution for these fractions, which, in comparison with other phases, form the following series: F3 (40.9) > F4 (25.2) > F2 (7.62) > F1 (2.44).

For Cd, the decreasing series of %F and CAF values are identical, that is, the result of CAF and %F approaches to estimate the chemical affinity for Cd is similar. For other HMs, the affinity series for CAF and %F differs. This is mainly manifested in a decrease in the role of F4 and an increase in the contribution of F3 for Cu, Mn, and Fe with the displacement of F4 in this series from the first place in %F to the second in CAF, and for Pb—from the second place to the third. For Pb and Zn, CAF shows a sharp increase in the contribution of F3 and a decrease in F2, for Co and Ni—an increase in F2 and a decrease in F1, for Cr—an increase in F3 and a decrease in F2 (Table 3). The biggest difference in the assessment of the HMs’ affinity to fractions by CAF and %F is that F3 becomes the leading fraction for Cu, Fe, Mn, Pb, and Cr when calculating CAF. Whereas F4 was the leading fraction for Cu, Fe, Mn and F2 was the leading fraction for Pb and Cr when calculating %F. That is, while assessing the chemical affinity of HMs by CAF compared to %F, a significant increase in the role of F3 is found. For atmospheric PM_10_ in Pune city, the ranking of elements by the CAF(∑F) value showed a decrease in contribution to the total pollution in the series: Co (11.3) > Cu (10.7) > Fe (10.2) > Mn (8.96) > Ni (8.50) > Cr (8.15) > Zn (7.52) > Pb (6.95) > Cd (3.79).

A full ranking of chemical fractions by CAF value showed that the highest pollution of PM_10_ of Pune city is formed by F3 Co (8.62) > F3 Cu (6.58) > F3 Fe (6.04) > F3 Mn (4.87) > F3 Cr (4.82) > F4 Zn (4.31) > F4 Ni (4.10) > F3 Ni (3.92) > F4 Fe (3.79) > F4 Cu (3.78) > F4 Mn (3.19) > F3 Pb (2.96) > F3 Zn (2.28) > F2 Pb (2.01) > F4 Co (2.00) > F2 Cr and F4 Pb (1.74) > F4 Cr (1.32). Other HM fractions have a less significant affinity for contamination with CAF < 1.2. The *r_s_* value between the series of CAF values for each fraction of each HM and the corresponding %F values is significant at *p* < 0.05 and amounts to 0.786, which indicates similarity in estimates of the chemical affinity of HMs for different geochemical phases in PM_10_ of Pune city using CAF and %F approach. At the same time, the Pearson’s correlation coefficient *r* between these values is also significant at *p* < 0.05 and amounts to 0.639, that is, the coefficient of determination *r*^2^ is 0.408. This may indicate that 40.8% of CAF variability is associated with a change in chemical affinity, while 59.2% can be associated with a change in pollution level.

Thus, the CAF and %F indices show a significant role of organic and residual fractions in the HMs accumulation; however, CAF additionally indicates a considerable increase in the role of the carbonate and bound to oxides fractions (especially for accumulation of Cu, Fe, Mn, Pb, and Cr). However, the calculation of *r_s_* between CAF and %F revealed close estimates of the chemical affinity of Cd, Cu, Zn, Fe, Mn, Co, Cr, and Ni using both indices. The CAF variability in PM_10_ by 40.8% is associated with a variation in chemical affinity.

### 3.5. Pollution Assessment of Particle Size Fractions of Road Dust with Chemical Fractions of HMs (GF-Analysis)

**Figure 4:**
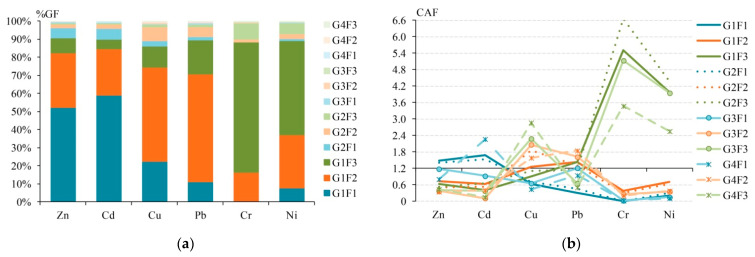
The fractionation distribution of HMs (**a**) and levels of chemical affinity factor (CAF) for HMs (**b**) for GF-fractions of road dust from Benowa suburbs, Gold Coast, South East Queensland, Australia—calculated from the data of A. Jayarathne et al. [100].

More detailed information on the fractionation of HMs in solid environmental media is provided by GF-analysis results, showing the distribution of chemical fractions of HMs in individual particle size fractions of soils, road dust, bottom sediments, and atmospheric particulate matter. CAF calculations for the results of GF-analysis were performed using the example of particle size fractions 0.45–75 μm (G1), 75–150 μm (G2), 150–300 μm (G3), and 300–425 μm (G4) of road dust in a commercial area in Benowa suburbs, Gold Coast, South East Queensland, Australia [100]. Zn, Cu, Pb, Cr, Ni, and Cd concentrations were studied in each G-fraction by the BCR sequential extraction scheme. Since in article [100] the results on the content of F-fractions are given per unit mass of each of the G-fractions, then for their conversion per unit mass of the entire road dust sample, data on the percentage of each G-fraction in road dust were used: 80.32% for G1, 11.54% for G2, 2.75% for G3, and 0.94% for G4 [104]. The following chemical fractions of HMs have been studied: F1—a sum of the exchangeable and carbonate fractions, F2—a sum of the reducible and oxidizable fractions, F3—residual fraction. The results of calculating the concentrations of GF-fractions in units of mass of road dust, the proportion of GF-fractions in the total content of each HM (%GF), and the CAF values for GF are shown in Figure 4 and Table 4.

Particles of the G1 fraction primarily represent the bulk road dust in Benowa suburbs. Therefore, the most significant contribution to the total content of all HMs is made by chemical fractions in these particles: %GF is maximal for G1F1 (F1 in particles 0.45–75 μm) for Zn and Cd, G1F2 (F2 in particles 0.45–75 µm) for Cu and Pb, G1F3 (F3 in particles 0.45–75 µm) for Cr and Ni (Figure 4). The predominance of the exchangeable and carbonate phases of Zn and Cd is typical for road dust [56]. In total, the three chemical fractions in G1 account for 86–91% of the total HMs content (Table 4). With an increase in the size of particles and a decrease in their contribution to the road dust material, their role in the total content of HMs formation decreases. In coarser particles, a noticeable contribution (%GF > 5%) is made by G2F1 for Zn and Cd, G2F2 for Cu and Pb, G2F3 for Cr and Ni, that is, the predominance of F1 for Zn and Cd, F2 for Cu and Pb, and F3 for Cr and Ni are typical for road dust particles of different sizes.

In general, the same pattern of HMs affinity to the indicated chemical fractions remains when calculating CAF. However, CAF defines a sharp increase in the degree of chemical affinity of Cu to the residual fraction, especially in coarse particles G3 and G4. In contrast, when using the %GF approach, F2 makes the main contribution to the Cu content in these particles. The second difference between CAF and %GF is the increased role of coarse particles in the individual HMs accumulation. Thus, the affinity of F1 of Cd to G4, Pb to G2, G3, and G4, and Cr to G3 and G4 is much more pronounced than the affinity of these HMs to G1. For Cu and Pb, the affinity of F2 to G2, G3, and G4 sharply increases compared with fine particles of G1. F3 is characterized by a higher affinity to G2 particles for Cd, Cr, and Ni and G4 for Cu rather than for G1 of these metals. Despite the indicated differences in the estimates of chemical affinity and affinity to particles using CAF and %GF, in general, the two approaches for most HMs give rather similar results. High and significant *r_s_* values confirm this for Zn (0.713), Cd (0.673), Cr (0.671), and Ni (0.580). The most different series of affinity for fractions when calculating CAF and %GF is revealed for Pb and Cu, for which *r_s_* is 0.189 and −0.175, respectively (Supplementary Materials Table S2). In general, cluster analysis results for the %GF and CAF are close: for both indices, the associations of Zn–Cd, Cu–Pb, and Cr–Ni are formed (Supplementary Materials Figure S1). However, %GF, in comparison with CAF, shows a closer clustering between individual HMs, while CAF indicates a closer association between HM groups.

The residual fraction F3 of Cr and Ni has an extremely strong affinity to road dust particles of any size (CAF > 2.4), and F3 of Cu still has an extremely strong affinity to particles 300–425 µm. At the same time, a clear chemical affinity (CAF 1.2–2.4) to 0.45–75 µm and 75–150 µm particles was defined for F1 of Zn and Cd, F2 of Cu and Pb, and F3 of Pb. A clear chemical affinity to particles of 150–300 µm was revealed for F1 and F2 of Pb, F2 and F3 of Cu.

To coarse particles of 300–425 µm, the clear affinity was revealed for F1 of Cd, and F2 of Cu and Pb. In turn, an extremely low affinity (CAF < 0.4) was noted for F1 and F2 of Cr and Ni in almost all particle size fractions; F1 of Pb in 0.45–75 µm particles; F2 of Zn and Cd and F3 of Cd in 150–300 µm particles; F2 of Zn and F3 of Cd in 300–425 µm particles. These findings indicate generally low participation of named GF-fractions in road dust pollution in Benowa suburbs. The ranking of elements by the CAF(∑GF) value showed a decrease in contribution to the total pollution in the series: Cr (22.0) > Ni (17.7) > Cu (16.4) > Pb (12.9) > Cd (9.68) > Zn (8.88). The significant role of the residual fraction in the contamination of all particle size fractions of road dust is confirmed by the high values of ∑CAF for all HMs in the GF-fractions, which form a series: G2F3 (14.5) > G1F3 (12.9) > G3F3 (12.8) > G4F3 (10.0) > G1F2 (5.15) > G2F2 (5.14) > G3F2 (4.81) > G4F2 (4.78) > G4F1 (4.61) > G2F1 (4.44) > G1F1 (4.37) > G3F1 (4.16).

The *r_s_* value between the series of CAF values for each GF-fraction of each HM and the corresponding %GF values is significant at *p* < 0.05 but not very high and amounts to 0.473. This indicates not such close trends in the change in CAF and %GF for different geochemical phases in road dust of Benowa suburbs. At the same time, the Pearson’s correlation coefficient *r* between these values is significant at *p* < 0.05 and amounts to 0.302, that is, the coefficient of determination *r*^2^ is 0.091, which may indicate that the CAF variability is weakly associated with the %GF variability (only 9.1% of the CAF variability is related to chemical affinity and affinity to particles). In comparison, 90.1% of CAF variability can be associated with changes in the pollution level.

Thus, the CAF index in comparison with the %GF index shows a sharp increase in the chemical affinity level of Cu to the residual fraction, especially in coarse particles G3 and G4; as well as an increase in the contribution of the coarse particles (G4, G3, and G2) to the accumulation of F1 fraction of Cd, Pb, and Cr, F2 fraction of Cu and Pb, and F3 fraction of Cd, Cr, Ni, and Cu. However, despite the revealed differences, the two approaches give similar results for Zn, Cd, Cr, and Ni. All HMs are characterized by a significant role of the residual fraction in contamination of all particle size fractions of road dust. The CAF variability in road dust of Benowa suburbs by 9.1% is associated with a variation in the chemical affinity and affinity to particles, which is almost two times less compared to the intensity of the affinity of HMs to the chemical fractions of agricultural soils in Dongdagou stream basin and bottom sediments of Daya Bay and four times less compared to the atmospheric PM_10_ of Pune city.

## 4. Conclusions

A complex CAF index is proposed to measure the chemical affinity of HMs to carrier phases and the contamination of solid environmental media with a chemical fraction of HMs. The strong point of CAF is the stability of the obtained HM series according to the degree of chemical affinity and contamination.

Comparison of CAF with %F calculated based on the literature examples on HMs’ fractionation in soils, bottom sediments, atmospheric PM_10_, and particle size fractions of road dust showed a more detailed assessment of the chemical affinity of HMs to individual geochemical phases in the CAF approach. CAF shows a less significant role of the residual fraction in the accumulation of HMs since CAF takes into account not only the chemical affinity but also the level of contamination with chemical fractions. Due to the normalization relative to the global geochemical reference, calculations of an individual element contribution to the total pollution by all studied HMs and contribution of the particular chemical fraction to the total HMs content; the CAF index shows a more significant role in pollution and chemical affinity of mobile and potentially mobile HMs’ forms. Thus, CAF shows a closer chemical affinity and a higher level of contamination of organic fraction of Cu, Pb, Cr, Ni, Zn, exchangeable fraction of Cd and reducible fraction of Mn in soils, acid-soluble and oxidizable fractions of most HMs in bottom sediments, and carbonate and bound to oxides fractions of Cu, Fe, Mn, Pb, Cr in atmospheric PM_10_. For the particle size fractions of road dust, CAF shows an increase in the coarse particles’ role in the accumulation of the exchangeable fraction of Cd, Pb, Cr, the reducible and oxidizable fraction of Cu and Pb, the residual fraction of Cd, Cr, Ni, Cu.

The proposed method does not have a specific suitable range threshold since any nonzero and positive values of the concentrations of the HMs chemical forms can be used for CAF calculation. We believe that the combined use of the proposed CAF index along with other standard indices allows a more detailed assessment of the affinity of individual HMs to certain geochemical phases, which is crucial for an understanding of the HMs’ fate in the environment and a more accurate evaluation of their mobility and bioavailability. Furthermore, the CAF approach is suitable for assessing differences in the fractionation of HMs within large spatial objects, for instance, megacities. This method will make it possible to identify critical pollutants for different cities and determine the chemical fractions that make the most significant contribution to the pollution.

Future empirical studies are required for a more precise assessment of chemical affinity and contamination of solid environmental media, taking into account the spatial distribution of HMs content. The aims of future CAF research may include: (1) Comparison of metal fractionation using CAF in one type of environmental compartment in different geographic conditions, or under different geochemical factors, or with different intensity of anthropogenic impact; and (2) the assessment of the environmental parameters’ impact (temperature, humidity, precipitation, pH value, content of organic matter, electrical conductivity, particle size distribution, etc.) on the CAF variability.

## Figures and Tables

**Table 1:** Results of HMs fractionation in agricultural soils from Dongdagou stream basin, China, and levels of chemical affinity factor (CAF)—calculated from the data of Y. Li et al. [86].

Parameter	Heavy Metals							∑CAF
	Cd	Ni	Pb	Zn	Cr	Cu	Mn	
F1, mg/kg	3.85	9.71	8.07	11.3	5.91	4.22	11.8	n/a
F2, mg/kg	4.17	16.2	60.5	108	1.16	40.0	85.6	n/a
F3, mg/kg	2.16	5.95	105	147	6.56	55.0	188	n/a
F4, mg/kg	0.28	5.94	48.8	60.9	5.79	123	37.2	n/a
F5, mg/kg	4.47	36.5	147	607	38.8	132	365	n/a
∑F, mg/kg	14.9	74.3	369	934	58.2	354	688	n/a
F1, % of ∑F	26	13	2	1	10	1	2	n/a
F2, % of ∑F	28	22	16	12	2	11	12	n/a
F3, % of ∑F	14	8	28	16	11	16	27	n/a
F4, % of ∑F	2	8	13	7	10	35	5	n/a
F5, % of ∑F	30	49	40	65	67	37	53	n/a
CAF(F1)	1.28	0.65	0.11	0.06	0.50	0.06	0.09	2.74
CAF (F2)	1.14	0.89	0.67	0.47	0.08	0.46	0.51	4.21
CAF (F3)	0.90	0.50	1.78	0.99	0.70	0.97	1.71	7.55
CAF (F4)	0.35	1.51	2.49	1.23	1.88	6.55	1.02	15.0
CAF (F5)	0.88	1.45	1.17	1.91	1.96	1.10	1.56	10.0
CAF (∑F)	4.55	4.99	6.22	4.66	5.13	9.14	4.88	39.6

Note. n/a—not available. F1 is exchangeable, F2 is carbonate, F3 is reducible (Fe-Mn oxyhydroxides), F4 is organic, F5 is residual fraction. CAF(∑F) is the sum of CAF for all fractions of a particular HM. ∑CAF is the sum of the affinity levels of all HMs of a specific chemical fraction.

**Table 2:** Results of HMs fractionation in surface sediments of Daya Bay and levels of chemical affinity factor (CAF)—calculated from the data of X. Gao et al. [111].

Parameter	Heavy Metals												∑CAF
	Ba	Cd	Co	Cu	Mn	Mo	Ni	Pb	Sc	Sr	U	Zn	
F1, mg/kg	0.91	0.01	0.82	0.49	408	0.01	1.91	2.16	0.04	144	0.07	2.22	n/a
F2, mg/kg	1.91	0.01	1.67	1.68	125	0.01	1.80	14.7	0.02	13.3	0.07	10.9	n/a
F3, mg/kg	2.24	0.01	1.20	1.05	50.5	0.14	3.19	1.43	0.19	1.75	0.59	6.12	n/a
F4, mg/kg	356	0.02	9.01	17.5	253	1.67	24.3	27.5	11.8	50.5	2.81	93.5	n/a
∑F, mg/kg	361	0.05	12.7	20.7	836	1.83	31.2	45.8	12.0	210	3.54	113	n/a
F1, % of ∑F	0	18	6	2	49	1	6	5	0	69	2	2	n/a
F2, % of ∑F	1	20	13	8	15	0	6	32	0	6	2	10	n/a
F3, % of ∑F	1	22	9	5	6	8	10	3	2	1	17	5	n/a
F4, % of ∑F	99	40	71	84	30	91	78	60	98	24	79	83	n/a
CAF(F1)	0.02	1.72	0.62	0.23	4.66	0.05	0.59	0.45	0.03	6.57	0.19	0.19	15.3
CAF(F2)	0.04	1.68	1.11	0.68	1.26	0.03	0.49	2.70	0.01	0.53	0.16	0.81	9.50
CAF(F3)	0.09	3.21	1.38	0.74	0.88	1.12	1.49	0.46	0.23	0.12	2.43	0.79	12.9
CAF(F4)	1.39	0.57	1.00	1.19	0.43	1.29	1.10	0.85	1.38	0.34	1.12	1.17	11.8
CAF(∑F)	1.55	7.18	4.10	2.84	7.23	2.49	3.66	4.45	1.66	7.56	3.90	2.96	49.6

Note. n/a—not available. Chemical fractions: F1—acid-soluble (exchangeable and bound to carbonates), F2—reducible (bound to Fe/Mn oxides), F3—oxidizable (bound to organic matter and sulfides), F4—residual (metals within lithogenic minerals) fraction. CAF(∑F) is the sum of CAF of all fractions of a particular HM. ∑CAF is the sum of the affinity levels of all HMs of a particular chemical fraction.

**Table 3:** Results of HMs fractionation in atmospheric PM_10_ of Pune city and levels of chemical affinity factor (CAF)—calculated from the data of R. Jan et al. [102].

Parameter	Heavy Metals									∑CAF
	Cu	Zn	Fe	Pb	Cd	Mn	Co	Cr	Ni	
F1, ng/m^3^	3.0	23	233	20	53	45	31	37	17	n/a
F2, ng/m^3^	16	59	375	66	20	62	21	94	7.0	n/a
F3, ng/m^3^	75	41	1699	24	4.0	106	105	64	27	n/a
F4, ng/m^3^	113	203	2795	37	12	182	64	46	74	n/a
∑F, ng/m^3^	207	326	5102	147	89	395	221	241	125	n/a
F1, % of ∑F	1	7	5	14	60	11	14	15	14	n/a
F2, % of ∑F	8	18	7	45	22	16	10	39	6	n/a
F3, % of ∑F	36	13	33	16	4	27	48	27	22	n/a
F4, % of ∑F	55	62	55	25	13	46	29	19	59	n/a
CAF(F1)	0.03	0.12	0.08	0.24	1.03	0.20	0.24	0.27	0.24	2.44
CAF(F2)	0.35	0.81	0.33	2.01	1.00	0.70	0.42	1.74	0.25	7.62
CAF(F3)	6.58	2.28	6.04	2.96	0.82	4.87	8.62	4.82	3.92	40.9
CAF(F4)	3.78	4.31	3.79	1.74	0.93	3.19	2.00	1.32	4.10	25.2
CAF(∑F)	10.7	7.52	10.2	6.95	3.79	8.96	11.3	8.15	8.50	76.1

Note. n/a—not available. Chemical fractions: F1—water-soluble, F2—environmentally mobile (exchangeable), F3—the sum of carbonate and bound to oxides fractions, F4—the sum of organic and residual fractions. CAF(∑F) is the sum of CAF of all fractions of a particular HM. ∑CAF is the sum of the affinity levels of all HMs of a particular chemical fraction.

**Table 4:** Results of chemical and grain size fractionation of HMs (GF-analysis) in road dust from Benowa suburbs, Gold Coast, South East Queensland, Australia, and levels of CAF —calculated from the data of A. Jayarathne et al. [100].

Parameter		Concentration, mg/kg of Road Dust					Parameter	Share in the Total Content, %					Parameter	CAF				
		G1	G2	G3	G4	∑G		%G1	%G2	%G3	%G4	∑%G		G1	G2	G3	G4	∑G
Zn	F1	540	56	4.8	1.5	602	%F1	52	5.4	0.47	0.15	58	F1	1.49	1.40	1.18	0.81	4.88
	F2	313	22	1.2	0.69	337	%F2	30	2.1	0.12	0.07	33	F2	0.73	0.51	0.37	0.35	1.97
	F3	87	7.6	0.41	0.38	95	%F3	8.4	0.7	0.04	0.04	9	F3	0.64	0.53	0.41	0.44	2.02
	∑F	939	86	6.5	2.6	∑GF = 1034	∑%F	91	8.3	0.63	0.25	∑%GF = 100	∑F	2.87	2.44	1.97	1.61	∑GF = 8.88
Cd	F1	0.44	0.04	0.003	0.003	0.49	%F1	59	6.0	0.37	0.41	66	F1	1.69	1.54	0.93	2.27	6.42
	F2	0.19	0.02	0.0003	0.001	0.21	%F2	26	2.2	0.04	0.08	28	F2	0.62	0.52	0.12	0.40	1.66
	F3	0.04	0.007	0.0003	0.0001	0.047	%F3	5.4	0.9	0.04	0.01	6	F3	0.41	0.66	0.38	0.15	1.60
	∑F	0.67	0.07	0.003	0.004	∑GF = 0.75	∑%F	90	9.1	0.44	0.50	∑%GF = 100	∑F	2.72	2.73	1.42	2.82	∑GF = 9.68
Cu	F1	61	7.7	0.72	0.22	70	%F1	22	2.8	0.26	0.08	25	F1	0.64	0.72	0.66	0.44	2.46
	F2	144	21	1.8	0.83	167	%F2	52	7.7	0.65	0.30	61	F2	1.26	1.87	2.07	1.59	6.78
	F3	33	4.3	0.61	0.67	39	%F3	12	1.5	0.22	0.24	14	F3	0.91	1.10	2.29	2.87	7.17
	∑F	238	33	3.1	1.7	∑GF = 276	∑%F	86	12.0	1.13	0.62	∑%GF = 100	∑F	2.81	3.69	5.02	4.90	∑GF = 16.4
Pb	F1	23	3.7	0.99	0.36	28	%F1	11	1.8	0.48	0.17	13	F1	0.31	0.46	1.22	0.95	2.94
	F2	123	11	1.1	0.72	136	%F2	60	5.4	0.51	0.35	66	F2	1.44	1.31	1.62	1.85	6.23
	F3	39	3.3	0.13	0.09	43	%F3	19	1.6	0.06	0.04	21	F3	1.44	1.15	0.66	0.51	3.76
	∑F	185	18	2.2	1.2	∑GF = 207	∑%F	90	8.8	1.06	0.56	∑%GF = 100	∑F	3.20	2.93	3.50	3.30	∑GF = 12.9
Cr	F1	0.48	0.05	0.008	0.004	0.54	%F1	0.7	0.1	0.01	0.01	1	F1	0.02	0.02	0.03	0.03	0.10
	F2	11	0.87	0.058	0.027	12	%F2	15	1.3	0.08	0.04	17	F2	0.38	0.31	0.27	0.21	1.16
	F3	50	6.4	0.34	0.20	57	%F3	72	9.3	0.50	0.29	82	F3	5.50	6.65	5.14	3.47	20.8
	∑F	61	7.3	0.41	0.23	∑GF = 69	∑%F	88	10.6	0.59	0.34	∑%GF = 100	∑F	5.90	6.97	5.43	3.71	∑GF = 22.0
Ni	F1	3.1	0.48	0.025	0.008	3.6	%F1	7.5	1.2	0.06	0.02	9	F1	0.22	0.30	0.15	0.11	0.78
	F2	12	1.1	0.047	0.030	13	%F2	30	2.6	0.11	0.07	32	F2	0.72	0.62	0.36	0.38	2.08
	F3	22	2.5	0.16	0.089	24	%F3	52	6.1	0.38	0.21	59	F3	3.97	4.39	3.95	2.56	14.9
	∑F	37	4.1	0.23	0.13	∑GF = 42	∑%F	89	9.9	0.56	0.31	∑%GF = 100	∑F	4.91	5.31	4.46	3.05	∑GF = 17.7
												Total	F1	4.37	4.44	4.16	4.61	17.6
													F2	5.15	5.14	4.81	4.78	19.9
													F3	12.9	14.5	12.8	10.0	50.2
													∑F	22.4	24.1	21.8	19.4	∑GF = 87.6

Note. G1 is 0.45–75 μm particles, G2 is 75–150 μm particles, G3 is 150–300 μm particles, G4 is 300–425 μm particles. F1 is effective bioavailable (sum of the exchangeable and carbonate fractions) fraction, F2 is potentially bioavailable (sum of the reducible and oxidizable fractions), F3 is non-bioavailable (residual) fraction.

## Data Availability

The data presented in this study are available on request from the corresponding author.

## Supplementary Materials

The following are available online at https://www.mdpi.com/article/10.3390/ijerph18168458/s1. Table S1: Indices to assess the heavy metal (HM) affinity to chemical fractions. Table S2: Spearman’s rank correlation coefficients (*r_s_*) between the CAF and %F values. Figure S1: Dendrograms of %F and CAF for HMs in soils (a,b), bottom sediments (c,d), atmospheric PM_10_ (e,f), and grain size fractions of road dust (g,h)—results of cluster analysis (amalgamation rule: complete linkage, distance measure: Euclidian distances).
