# Supplementary material for: Integrated Assessment of Affinity to Chemical Fractions and Environmental Pollution with Heavy Metals: A New Approach Based on Sequential Extraction Results
Source: Int J Environ Res Public Health. 2021 Aug 10;18(16):8458. doi: 10.3390/ijerph18168458 (PMC8391145; doi:10.3390/ijerph18168458)
Supplement: Supplementary file 1 [file ijerph-18-08458-s001.zip › ijerph-1266295-supplementary.pdf]

International Journal of
Environmental Research and Public Health

MDPI

Supplementary Materials to:

Integrated Assessment of Affinity to Chemical Fractions and Environmental Pollution with Heavy Metals: A New Approach Based on Sequential Extraction Results

Yuri Vodyanitskii and Dmitry Vlasov

Table S1. Indices to assess the heavy metal (HM) affinity to chemical fractions.

Index	Equation	References
Percentage of fraction (%F), percent of the sum (PFS)	$\%F = PFS = 100\% \times F_i / C_{total}$	[86]
Individual contamination factor (ICF)	$ICF = (C_{total} - C_{res}) / C_{res}$	[87]
Risk assessment code (RAC)	$RAC = \%F1 + \%F2$	[93]
Mobility factor (MF)	$MF = \%F1 + \%F2 + \%F3$	[88]
Bioavailability factor (BF)	$BF = \%F1 + \%F2 + \%F3 + \%F4$	[89]
Mobility coefficient (MC)	$MC = LB/SB$	[90]
Stability coefficient (SC)	$SC = 100\% \times C_{res} / SB$	[90]
Protecting coefficient (PC)	$PC = 100\% - (C_{mob}/C_{total}) \times 100\%$	[91]
Anthropogenic signal index (ASI)	$ASI = (C_{part} / C_{tot})_n / (C_{part} / C_{tot})_{Al}$	[92]
Bioavailable metal index (BMI)	$BMI = \left(\frac{C_{F1}^{1}}{C_{B-F1}^{1}} \times \frac{C_{F1}^{1}}{C_{B-F1}^{1}} \times \ldots \times \frac{C_{F1}^{n}}{C_{B-F1}^{n}}\right)^{1/n}$	[94]
Global contamination factor (GCF)	$GCF = \sum ICF$	[87]
Global risk index (GRI)	$GRI = \sum (Tr \times ICF)$	[70]

Note. C_{total} is the total HM content, C_{res} is the HM content in the residual fraction, C_{mob} is the content of mobile HM forms (usually extractable with acids, that is, the content of the acid-soluble HM fraction), %F1 is the percentage of the water-soluble and exchangeable HM fractions, %F2 is the percentage of the carbonate HM fraction, %F3 – the same for the reducible HM fraction, %F4 – for the organic HM fraction, LB is the content of loosely bound (or mobile) HM compounds (e.g., HMs retained on the organic and mineral soil components surface in an exchangeable or specifically sorbed state), SB is the content of strongly bound HM compounds (e.g., HMs retained by organic substances, iron (hydr)oxides, and silicates), n is the number of analyzed chemical elements, C_{F1}^{1} and C_{B-F1}^{1} are of contents sum of exchangeable and carbonate fractions (in the BCR method corresponds to the F1 fraction) of a chemical element in the study object and the background analogue, respectively; Tr is the "toxic-response" factor according to L. Håkanson [74] for an individual chemical element. For ASI, the numerator is the ratio of the leached form of the element n concentration (C_{part}) to the total content of the element n, the denominator is the ratio of the same indicators for aluminum.

Table S2. Spearman's rank correlation coefficients (r_s) between the CAF and %F values.

HMs	Soils [86]	Bottom sediments [111]	Atmospheric PM_{10} [102]	Particle size fractions of road dust [100]
Cd	0.300	–0.400	1.000*	0.673*
Ni	0.000	0.800	0.800	0.580*
Pb	0.300	0.600	0.400	0.189
Zn	0.700	1.000*	0.800	0.713*
Cr	0.700	N/A[1]	0.800	0.671*
Cu	0.900*	0.800	0.800	–0.175
Mn	0.800	0.400	0.800	N/A
Ba	N/A	1.000*	N/A	N/A
Co	N/A	0.200	0.800	N/A
Mo	N/A	1.000*	N/A	N/A
Sc	N/A	1.000*	N/A	N/A
Sr	N/A	0.800	N/A	N/A
U	N/A	0.800	N/A	N/A
Fe	N/A	N/A	0.800	N/A

[1] N/A – not available. Values significant at $p < 0.05$ marked with "*".

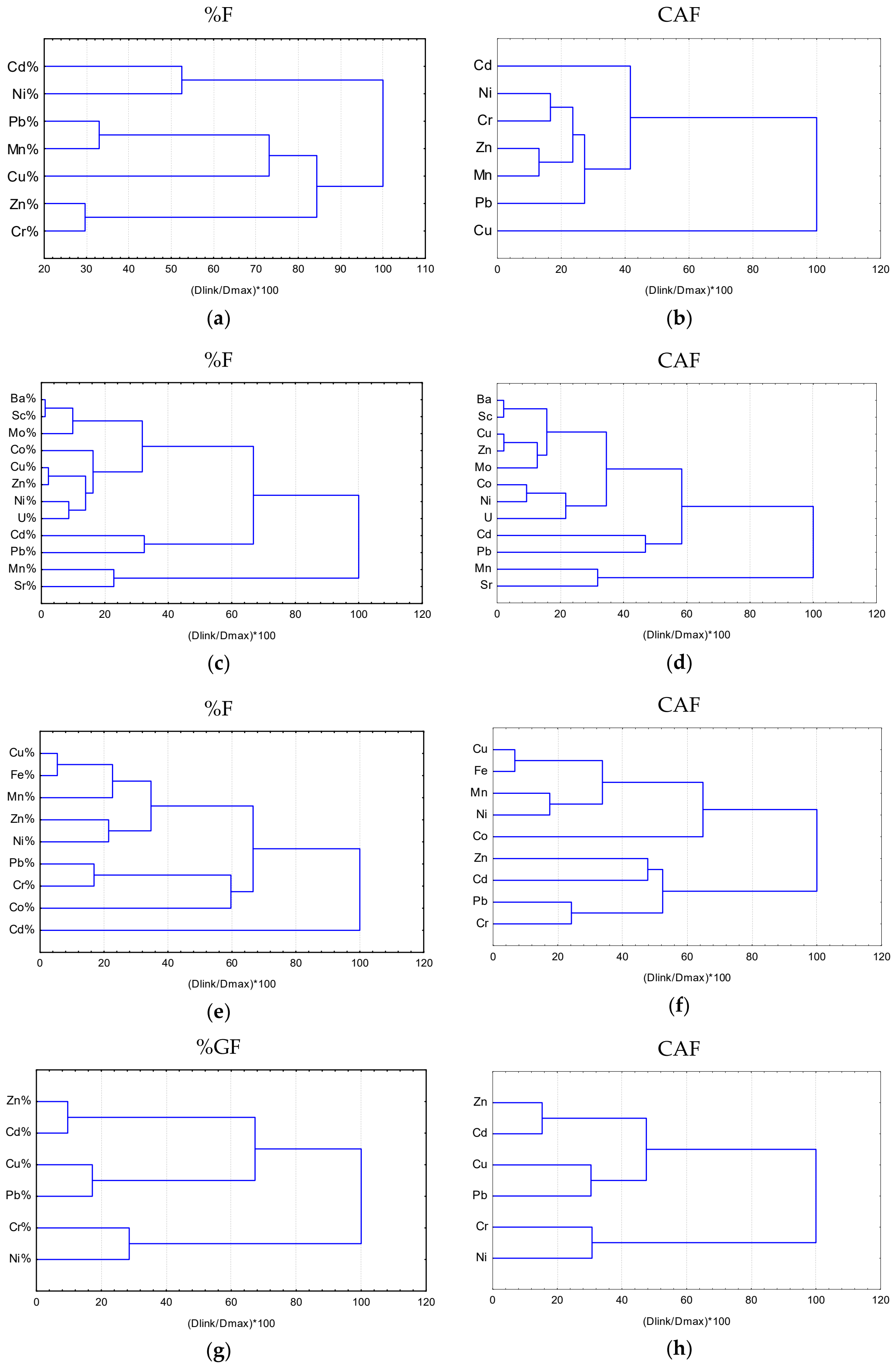

Figure S1. Dendrograms of %F and CAF for HMs in soils (**a**, **b**), bottom sediments (**c**, **d**), atmospheric PM_{10} (**e**, **f**), and grain size fractions of road dust (**g**, **h**). Results of cluster analysis (amalgamation rule: complete linkage, distance measure: Euclidian distances).

References

86. Li, Y.; Wang, S.; Nan, Z.; Zang, F.; Sun, H.; Zhang, Q.; Huang, W.; Bao, L. Accumulation, Fractionation and Health Risk Assessment of Fluoride and Heavy Metals in Soil-Crop Systems in Northwest China. *Sci. Total Environ.* **2019**, *663*, 307–314, doi:10.1016/j.scitotenv.2019.01.257.
87. Ikem, A.; Egiebor, N.O.; Nyavor, K. Trace Elements in Water, Fish and Sediment from Tuskegee Lake, Southeastern USA. *Water Air Soil Pollut.* **2003**, *149*, 51–75, doi:10.1023/A:1025694315763.
93. Perin, G.; Craboledda, L.; Lucchese, M.; Cirillo, R.; Dotta, L.; Zanetta, M.L.; Oro, A.A. Heavy metal speciation in the sediments of northern Adriatic Sea. A new approach for environmental toxicity determination. In *Heavy Metals in the Environment*; Lakkas, T.D., Ed.; CEP Consultants: Edinburgh, Scotland, 1985; Volume 2, pp. 454–456.
88. Ghrefat, H.A.; Yusuf, N.; Jamarh, A.; Nazzal, J. Fractionation and Risk Assessment of Heavy Metals in Soil Samples Collected along Zerqa River, Jordan. *Environ. Earth Sci.* **2012**, *66*, 199–208, doi:10.1007/s12665-011-1222-6.
89. Kidd, P.S.; Domínguez-Rodríguez, M.J.; Díez, J.; Monterroso, C. Bioavailability and Plant Accumulation of Heavy Metals and Phosphorus in Agricultural Soils Amended by Long-Term Application of Sewage Sludge. *Chemosphere* **2007**, *66*, 1458–1467, doi:10.1016/j.chemosphere.2006.09.007.
90. Mandzhieva, S.S.; Minkina, T.M.; Motuzova, G.V.; Golovatyi, S.E.; Miroshnichenko, N.N.; Lukashenko, N.K.; Fateev, A.I. Fractional and Group Composition of Zinc and Lead Compounds as an Indicator of the Environmental Status of Soils. *Eurasian Soil Sci.* **2014**, *47*, 511–518, doi:10.1134/S1064229314050159.
91. Karpukhin, A.I.; Bushuev, N.N. Effect of fertilization on the content of heavy metals in soils of long-term field experiments. *Agrokhimia* **2007**, *5*, 76–84.
92. Sutherland, R.A.; Tack, F.M.G.; Ziegler, A.D.; Bussen, J.O. Metal Extraction from Road-Deposited Sediments Using Nine Partial Decomposition Procedures. *Appl. Geochem.* **2004**, *19*, 947–955, doi:10.1016/j.apgeochem.2003.11.002.
94. Rosado, D.; Usero, J.; Morillo, J. Assessment of Heavy Metals Bioavailability and Toxicity toward Vibrio Fischeri in Sediment of the Huelva Estuary. *Chemosphere* **2016**, *153*, 10–17, doi:10.1016/j.chemosphere.2016.03.040.
70. Zhao, S.; Feng, C.; Yang, Y.; Niu, J.; Shen, Z. Risk Assessment of Sedimentary Metals in the Yangtze Estuary: New Evidence of the Relationships between Two Typical Index Methods. *J. Hazard. Mater.* **2012**, *241–242*, 164–172, doi:10.1016/j.jhazmat.2012.09.023.
74. Hakanson, L. An Ecological Risk Index for Aquatic Pollution Control.a Sedimentological Approach. *Water Res.* **1980**, *14*, 975–1001, doi:10.1016/0043-1354(80)90143-8.
111. Gao, X.; Chen, C.-T.A.; Wang, G.; Xue, Q.; Tang, C.; Chen, S. Environmental Status of Daya Bay Surface Sediments Inferred from a Sequential Extraction Technique. *Estuar. Coast. Shelf Sci.* **2010**, *86*, 369–378, doi:10.1016/j.ecss.2009.10.012.
102. Jan, R.; Roy, R.; Yadav, S.; Satsangi, P.G. Chemical Fractionation and Health Risk Assessment of Particulate Matter-Bound Metals in Pune, India. *Environ. Geochem. Health* **2018**, *40*, 255–270, doi:10.1007/s10653-016-9900-7.
100. Jayarathne, A.; Egodawatta, P.; Ayoko, G.A.; Goonetilleke, A. Assessment of Ecological and Human Health Risks of Metals in Urban Road Dust Based on Geochemical Fractionation and Potential Bioavailability. *Sci. Total Environ.* **2018**, *635*, 1609–1619, doi:10.1016/j.scitotenv.2018.04.098
